# Editorial: Sex differences and sex steroid effects on musculoskeletal health

**DOI:** 10.3389/fendo.2022.991989

**Published:** 2022-09-13

**Authors:** Abdul Malik Tyagi, Sadiq Umar

**Affiliations:** ^1^ Division of Endocrinology, Council of Scientific And Industrial Research-Central Drug Research Institute (CSIR-CDRI) Lucknow, Uttar Pradesh, India; ^2^ Department of Oral Biology, College of Dentistry, University of Illinois, Chicago, IL, United States

**Keywords:** sex steroids, osteoblast, osteoclast, osteoporosis, bone mass

This Research Topic published original research articles and reviews from both human studies and animal models to discuss the effect of sex steroid hormones on musculoskeletal health. A total of 11 articles were included in this Research Topic and were divided into four categories: (1) original articles from *in vitro* studies, (2) original articles from animal studies, (4) original articles from human studies, and (4) review articles. Out of these 11 articles, one article was an *in vitro* study, four articles were from studies in animal models of bone loss, five articles were from human studies, and two were review articles. The role of sex steroid hormones on bones has been extensively studied. Estrogen deficiency-induced bone loss in women is defined as primary osteoporosis. There are several mechanisms involved in bone loss of sex steroid–deficient conditions such as increased reactive oxygen species, increased osteoclastogenesis and life span of osteoclast, increased osteoblast apoptosis, and immune system activation. However, in men, it is the testosterone (T) hormone that regulates bone mass. Even though we have ample information about the pathophysiology of osteoporosis and its treatment, this Research Topic provides novel insights into the role of sex steroid hormones on musculoskeletal health.

## Article from *in vitro* study

Wisanwattana et al. showed that prenylated flavonoid 14 promotes osteoblast differentiation by activating the cGMP/PKG/SHP2/Src/ERK cascade *via* phosphodiesterase 5 (PDE5) inhibition, thereby leading to the localized production of estrogen by stimulating aromatase expression. This study provides new insights into the use of PDE5-inhibiting drugs to mimic the anabolic effects of mechanical bone stimulation in the treatment of osteoporosis.

## Articles from animal studies

Saul et al. studied the effect of two lipoxygenase inhibitors, baicalein and zileuton, on ovariectomy (OVX)-induced bone loss in female rats and showed that the oral administration of baicalein did not improve either the vertebra or the femur. Zileuton showed a favorable effect on the trabecular vertebra, while the femur was negatively affected. Deng et al. utilized bioinformatics tools to study the mechanism of estrogen deficiency-induced bone loss. They observed 38 downregulated and 30 upregulated differentially expressed genes (DEGs). Through gene ontology analysis, the authors found that downregulated DEGs were mainly enriched in myeloid cell differentiation and cytokine-related functions, while upregulated DEGs were enriched in immune-related biological processes, pathways like Notch signaling and mitogen-activated phosphate kinase activation.

Zhou et al. studied the estrogenic effect of water extract of *Rhizoma Drynariae* (RD) administered with tamoxifen. The authors showed the interactions between RD and tamoxifen in the bone, brain, and uterus of OVX rats, while RD did not alter their responses to tamoxifen. The study further revealed that RD selectively exerts estrogenic actions differently from tamoxifen. Moreover, RD interacts with tamoxifen without altering its effects in OVX rats. Zhou et al. studied the effect of a single or combined administration with parathyroid hormone (PTH) and zoledronate acid (ZOL) on implant loosening in a rat model of osteoporosis. In this study, the authors showed that the combined treatment and monotherapy of PTH and ZOL enhanced the periprosthetic bone volume and bone–implant contact and the intramedullary implant stability in a debris wear-induced periprosthetic osteolysis under a condition of osteoporosis. Moreover, the combined PTH and ZOL therapy revealed an additive effect on preventing periprosthetic osteolysis and improving prosthetic anchorage, exhibiting a greater improvement than monotherapy that was even similar to or higher than that of the normal control group. Their findings indicate that combination or monotherapy with PTH and/or ZOL might be a promising strategy for preventing early-stage implant loosening in patients with severe osteoporosis.

## Articles from human studies

Kim et al. studied the effect of tissue-specific estrogen complex (TSEC) treatment on hip geometry in postmenopausal women from Korea. They reported improvement in bone geometry in postmenopausal women after 12 months of treatment with TSEC, and it can be utilized for the prevention of fracture as well as osteoporosis in postmenopausal women. As sex steroids are thought to play a critical role in the pathogenesis of osteoarthritis (OA), Yan et al. studied the effect of sex steroids on site- and sex-specific OA and the risk of joint replacement surgery using the Mendelian randomization (MR) method. Their data showed a positive causal association between serum T levels and risks of hip OA. Serum dihydrotestosterone level was also positively associated with the risk of hip replacement. Horwath et al. examined the effect of moderate-dose T administration on molecular regulators of muscle protein turnover and mitochondrial remodeling in muscle samples collected from young women. The authors found that the improvements in muscle size and oxidative capacity in young women in response to moderate-dose T administration cannot be explained by alterations in the total expression of molecular factors known to regulate muscle protein turnover or mitochondrial remodeling. Xu et al. assessed the association of bone mineral density (BMD) with sex hormones (including T and estradiol) and sex hormone-binding globulin (SHBG) in adolescent boys and girls aged 12–19 years. Based on these findings, an appropriate increase in serum testosterone levels may be beneficial for skeletal development in girls because of the inverted U-shaped relationship (with the inflection point at 25.4 ng/dl of testosterone), and a high testosterone level might be detrimental to BMD. Furthermore, keeping the estradiol levels below a certain level in boys (24.3 pg/ml) may need to be considered.

## Review articles

Another interesting study by Lin et al. reviewed the relationship between breast cancer and osteoporosis. They concluded that neuropeptide Y and its associated factors play a vital role in the development of osteoporosis and breast cancer and can be a novel diagnostic and therapeutic target for osteoporosis and breast cancer. Parker et al. reviewed the current evidence regarding the impact of relaxin on the incidence of soft tissue hip injuries in women. They found that at molecular levels, relaxin activates matrix metalloproteinases (MMPs) including collagenases MMP-1/-13 and gelatinases MMP 2/-9 to loosen pelvic ligaments for parturition. The authors concluded that menstrual cycle peaks of relaxin activate MMPs, which locally degrade collagen and gelatin. Women have relaxin receptors in multiple joints, including the hip and knee, and increased relaxin correlates with increased musculoskeletal injuries. Relaxin has paracrine effects in the female pelvis on ligaments adjacent to hip structures, such as acetabular labral cells, which express high levels of relaxin-targeted MMPs.

This Research Topic covers many important aspects of sex steroids and skeletal health with current updates and therapeutic strategies, which provide a better understanding of the disease diagnosis and treatment options in musculoskeletal health.

## Author contributions

AT and SU wrote the manuscript. AT and SU corrected and approved the final manuscript. All authors contributed to the article and approved the submitted version.

## Conflict of interest

The authors declare that the research was conducted in the absence of any commercial or financial relationships that could be construed as a potential conflict of interest.

## Publisher’s note

All claims expressed in this article are solely those of the authors and do not necessarily represent those of their affiliated organizations, or those of the publisher, the editors and the reviewers. Any product that may be evaluated in this article, or claim that may be made by its manufacturer, is not guaranteed or endorsed by the publisher.

